# Maternity care and Human Rights: what do women think?

**DOI:** 10.1186/s12914-016-0091-1

**Published:** 2016-07-02

**Authors:** Andrea Solnes Miltenburg, Fleur Lambermon, Cees Hamelink, Tarek Meguid

**Affiliations:** Department of Community Medicine, Institute of Health and Society, University of Oslo, Oslo, Norway; Athena Institute for Research on Innovation and Communication in Health and Life Sciences, VU University Amsterdam, Zanzibar Town, The Netherlands; Department of Obstetrics & Gynaecology, Mnazi Mmoja Hospital, Stone Town Zanzibar, Tanzania

**Keywords:** Human rights, Maternal health, Dignity

## Abstract

**Background:**

A human rights approach to maternal health is considered as a useful framework in international efforts to reduce maternal mortality. Although fundamental human rights principles are incorporated into legal and medical frameworks, human rights have to be translated into measurable actions and outcomes. So far, their substantive applications remain unclear. The aim of this study is to explore women’s perspectives and experiences of maternal health services through a human rights perspective in Magu District, Tanzania.

**Methods:**

This study is a qualitative exploration of perspectives and experiences of women regarding maternity services in government health facilities. The point of departure is a Human Rights perspective. A total of 36 semi-structured interviews were held with 17 women, between the age of 31 and 63, supplemented with one focus group discussion of a selection of the interviewed women, in three rural villages and the town centre in Magu District. Data analysis was performed using a coding scheme based on four human rights principles: dignity, autonomy, equality and safety.

**Results:**

Women’s experiences of maternal health services reflect several sub-standard care factors relating to violations of multiple human rights principles. Women were aware that substandard care was present and described a range of ways how the services could be delivered that would venerate human rights principles. Prominent themes included: ‘being treated well and equal’, ‘being respected’ and ‘being given the appropriate information and medical treatment’.

**Conclusion:**

Women in this rural Tanzanian setting are aware that their experiences of maternity care reflect violations of their basic rights and are able to voice what basic human rights principles mean to them as well as their desired applications in maternal health service provision.

**Electronic supplementary material:**

The online version of this article (doi:10.1186/s12914-016-0091-1) contains supplementary material, which is available to authorized users.

## Introduction

Sub-standard quality of care during pregnancy and childbirth is increasingly acknowledged to negatively influence subsequent care seeking and an important underlying cause for maternal morbidity and mortality [1–8]. Quality of care, however, is a complex concept often defined through an interaction of structure aspects of the health system, process of care provision including technical and interpersonal aspects and outcomes of care [9, 10]. Recent years increased attention has been given to interpersonal quality of care with concerns about maltreatment during childbirth [11]. Numerous authors report abusive language of health care workers (HCWs), rudeness, intimidation, scolding, lack of empathy, lack of privacy, uninformed decision making, physical assault and denial of services [4–6, 8, 12]. Such disrespectful treatment implies that multiple human rights principles are violated, in particular the right of every woman to be treated with respect for their dignity [13, 14].

‘The Respectful Maternity Care Charter: the Universal Rights of Childbearing Women’, launched by the White Ribbon Alliance, is a recent attempt to clearly demonstrate the link of human rights in maternal health with focus on the interpersonal aspects of care received by women seeking maternity services [15]. Applying such a human rights based approach to maternal health requires attention to the fundamental human rights principle of dignity as well as its related principles autonomy, equality and safety which are relevant in all kinds of health care settings [16]. Although these fundamental principles are incorporated into several legal and medical instruments, their implication and meaning ‘on the ground’ (for example, in the labour room) are far from clear [17–20]. Anecdotes of disrespect and abuse in health facilities, as descriptions of violations of these principles, is an attempt to operationalize human rights violations during childbirth but does not capture the complex interaction of experiences of women, characteristics of health provider behaviour and environmental factors that together influence quality of care [2, 21, 22]. At the same time, assessment of human rights violations alone is limited to what care should ‘not’ be, rather than understanding how care could be provided to ensure human rights principles are respected.

It is believed, that a lack of information about health rights during pregnancy and childbirth, influences women’s perception on maternal health services and an apparent passive acceptance of substandard quality of care [5, 6, 20, 23]. Relying on women’s experiences might therefore be limited due to assumed normalization of sub-standard care in the health system [14, 21]. Nevertheless, any attempt to understand complex quality of care concepts should start with analysis of ‘what women need and want’ [21]. Therefore the aim of this study is to understand women’s perceptions and experiences of maternal health services from a human rights perspective in a low-resource setting in Magu district, Tanzania.

## Background

Quality of care provided, and in particular the interpersonal component of quality of care, during pregnancy and childbirth should ensure that women are treated with respect for their ‘dignity’. The Universal Declaration of Human Rights (UDHR) placed dignity at the center of its description of basic human rights and as a consequence the concept of dignity has universally been adopted to be a fundamental principle of human rights [17]. Attention needs to be given to claim of critics that the vagueness of the term hinders its effective implementation [24]. Jacobsen argues this vagueness is largely attributed to lack of distinguishing ‘human dignity’ from ‘social dignity’ [24]. Where human dignity refers to a person’s ability to make moral choices [25–27], social dignity refers to a person’s position in society and social interactions with others [24, 27]. Following this distinction, it can be argued that sense of dignity is susceptible to internal and external actions [28].

Within healthcare, considerations of dignity as a concept have primarily been confined to the fields of palliative care and elderly care [25, 26]. In maternal health, dignity, as a human rights principle, has developed most fully within the context of family planning services with ‘the right to full information and freedom of choice for contraceptive method’ [29]. When dignity concepts are applied to women in labour the same rights apply but are often clouded by other priorities. Women in labour are at their most vulnerable, often fully depended on the health provider and the surrounding health system. Access to evidence-based care, provided with respect for the woman and her child’s dignity, including informed decision-making should be at the center of the provision of care. Far too often however, the technical aspects of care during labour are prioritised beyond the ways in which services are provided. Attributes of what ‘ dignity’ looks like and how it is experienced in a clinical setting remains poorly explored [13, 14].

### Dignity

In our evaluation of dignity we distinguish three dimensions of dignity; dignity in person, dignity in relation and dignity in institutions [25, 26]. The personal dimension of dignity is associated with one’s perception of worthiness as an individual and autonomous human being [25]. Based on this, the meaning of dignity can be simplified to equal ‘worthiness’ [14]. The personal dimension of dignity is subjective and culturally influenced by a person’s environment increasing the complexity with operationalization of dignity as a concept [26, 27]. Social relationships and interactions between individuals and groups can promote or hinder a person’s sense of dignity [25, 30]. According to Jacobson [26], relational dignity refers to individual and collective behaviour towards a person in terms of respect and worth. As interactions take place in a certain context, the environment and institutional structures can influence such “dignity encounters” [26]. For example, some settings such as health or law institutions accord individuals or groups authority to dominate others [31]. Understanding the practical implication of these three dimensions requires some elaboration on the principles of autonomy, equality and safety. These principles are heavily interrelated and interdependent, as dignity cannot be respected, without respect for the other three principles.

### Autonomy

Autonomy includes having the ability to participate in decision-making and being able to effectively project a voice [30, 32, 33]. Autonomy is associated with a lack of dependence on others, being self-reliant [33, 34] and exercising choice and in doing so becoming self-determinant. In health care settings this is enacted in patients right to make their own (informed) choices [33, 35]. This again is highly contextual and expressed differently across cultures. Inherent to self-determination is informed consent, which ensures that people make informed decisions consistent with what they perceive as being in their best interests [35, 36].

### Equality

Equality is defined as ‘the state of being equal’ [37] and applies to all rights and principles of the UDHR. Peoples circumstances are however not equal, which do not allow for equal opportunities or equal access. While equality is easily explained to be ‘sameness’, equity is referred to as ‘fairness’. Equity is the way to strive for equality in health, including the social determinants of health [38]. Health inequity therefore is the failure to overcome barriers which are caused by inequalities [39–41]. Additionally access to health care should be on the basis of non-discrimination regarding sex, age, ethnicity, socioeconomic status, religion or otherwise.

### Safety

Safety and security are definitions which are often used interchangeable and refer to respectively; protection from unintentional and intentional harm [42, 43]. Safety measures need to be in place in health facilities. Although healthcare services aim for health promotion and delivering life saving measures, receiving treatment is not without risk. Key contributors to unsafe care are a lack of health professionals, miscommunication among health professionals and lack of experience [44]. In addition, organisational aspects such as insufficient and inappropriate equipment and supplies can also lead to hazardous situations [45, 46].

We will evaluate women’s perception and experiences of maternal health services, against the conceptual elements of human rights principles as described above. Exploring human rights principles is a highly complex and contextually determined experience, nevertheless, we believe descriptions elicited from the women in this study provide some insight into the ways in which ‘dignity’ is perceived and experienced when receiving maternal health services in Magu District. It must be recognized that in a health care setting, health provider’s rights are as important as the rights of women who receive services. Discussing dignity from the woman’s perspective is not meant to point fingers at their providers without acknowledging constrains of the system in which they work [47].

## Methods

### Study setting

Data collection took place in Magu District, Tanzania over 12 weeks between April and July 2013. Magu District is one of eight districts of the Mwanza region in Northwestern Tanzania with a population of approximately 300,000 citizens. The majority of the people belong to the Sukuma tribe, which is the largest ethnic group of Tanzania [48]. The focus was on childbirth experiences.

### Data collection

In-depth interviews (*N* = 36) were held with 17 women between the ages of 31 and 63. Women were recruited in four different geographical locations in the district with help of the village executive officer to respect existing hierarchical structures. Key inclusion criteria were fluency in Kiswahili, minimum age of 30 and history of three or more facility based childbirth experiences. Women were included in a higher age group as they were expected to have more childbirth and life experiences on which to base their perceptions.

The duration of an interview varied from 25 min to 2 h and took place in a woman’s home. Acknowledging the difficulty and sensitivity of the topic, multiple interviews were held with each participant to increase comfort and ease of expression and to facilitate reflection on previous statements during follow-up interview. Most women were interviewed twice. One woman was interviewed only once as she cancelled the second interview for an unknown reason. Two women were interviewed three times and four times as new themes emerged during interviews, which required further elaboration.

Follow-up interviews enabled women to engage in an exploration of their perceptions about human rights principles in relation to provision of maternal health services. Women were encouraged to give examples or share a personal experiences. During the interviews it appeared to be easier for women to explain situations where they felt human rights principles were violated. After all interviews and a preliminary analysis, a focus group discussion, comprising five of the interviewed women, was held in one of the villages. During this FGD, women were invited to verify preliminary findings, including attributes of the four human rights principles and causes of their violations as presented by the women in the interviews. Additionally they were asked to share suggestions for improvements of health services during an open discussion.

A Dutch Public Health student who did not speak fluent Kiswahili held the interviews and therefore a research assistant translated both during interviews and the FGD. The research assistant, fluent in both English and Kiswahili and born in the research area assisted in preparing interviews and in developing the semi-structured interview guideline (see Additional file 1). To ensure all human rights principles were well understood within the context and in Kiswahili the guideline was translated into Kiswahili and back into English and included several discussions with the research team. It was important to ensure difficult concepts would not get lost in translation, in particular because these concepts are not part of daily language use. Sometimes examples, metaphors or local stories were needed to exemplify terminology used, often in relation to cultivation or domestic activities and household relations.

### Data analysis

All interviews and the FGD were recorded and transcribed into English. A few interviews were first analysed using open coding to gradually make sense of the data. Based on this, a comprehensive coding scheme with several categories, incorporating the pre-determined human rights principles, was developed for analysis. New codes were developed along the way if women’s accounts or experiences that emerged from the interviews did not fit in the coding scheme. Coding of all transcripts was done with a qualitative data analysis software programme (MAXQDA version 11).

## Results

Women’s general understanding of human rights in relation to facility based maternity services embraced the following: provision of personal care by a HCW and availability of diverse medical and non-medical equipment inclusive of beds, blood transfusion and medicine. Furthermore, women believed that such services should be timely and guarantee a certain level of safety. General perceptions about ‘human rights’ captured the notion of ‘being treated well’ which was explained as ‘receiving the services that you need’.

*‘For example, when you go to a health facility and you have a problem, you don’t have enough water or blood… Human rights mean that you go there and you receive good and fast services’* (Participant, Mahaha Village).

According to other women, human rights were also perceived in the following terms ‘to be independent’, ‘not to be humiliated’ and ‘to be treated equally’.

## Being treated well—Dignity

For most women ‘being treated well’ by HCWs implied being taken care of during and after birth. Examples cited included being comforted or being washed with hot water. Many women stated that ‘being treated well’ needs to begin with ‘being *received* well’. Conceptually, ‘being *received* well’ embraced a number of procedures upon arrival at the facility, physical examination and being informed about the process of labour. One woman clearly explained that patients feel relieved and comforted when received with a smile and calm polite words.

*‘Hospitality is the way you receive people. You receive with a smile and through words. Hospitality is not about giving things to people. You use polite language. […] When you talk to patients in a calm way, it can be a relief to them’* (Participant, Mahaha Village).

Location and events at the health facility seem to contribute to women’s perceptions of being ‘treated well’. Women reported being frightened by behaviour of other women such as witnessing struggling movements, and talked about how shy they felt when having to undress in front of other women. Furthermore, they voiced a fear of being teased by other women for their own behaviour, such as crying, during deliver.

*‘Women come and start to afraid others, they just want to make noise, they cry. So, they can cause others to be afraid. So, it is better if you are in one room with a doctor and no one is seeing you’* (Participant, Matale Village).

Women stated that HCWs can treat you well by taking care of you, which they explained as ‘receiving comfort’. HCWs can comfort a woman by using comforting words, giving tips and exercises to ease the labour pain. One woman indicated that she felt comforted by HCWs who were singing a song to her, which also motivated her to come back the next time.

*‘Some nurses they comfort you, they direct you how to push, and they sing a song for you. […] They comfort you in a good way. Let’s pray to God and you are going to deliver safely. When in the end you deliver safely, next time you will be motivated to come because the nurse was very nice, she can help you’ (Participant, Mahaha Village).*

Conceivably of ‘not being treated well’ and not respected is the experience of indignity or humiliation. The experience of humiliation, was expressed as follows:

*‘Humiliation is somehow that you don’t receive the services or the needs that you are looking for’ (*Participant, Matale Village). ‘*There is something you have to receive, and then you are hindered [by someone] to receive it’ (Participant, Mahaha village).*

Violations of the principle of dignity covered examples including the other three human rights principles and were mainly related to interactions with HCWs. Examples included, women being scolded at by HCWs for not having enough money, not bringing medical equipment, not being dressed properly, or for no apparent reason. In addition to verbal assaults, women also experienced physical abuse. Women were slapped during labour when they did not agree with the HCW or when they did not follow the instructions.

*‘They say: ‘We are not the one who have told you to get that pregnancy, shenzi [barbarian]. So push!! You only prefer for yourself to get pregnant, not for us.’ […] They are humiliating. You should not humiliate your fellow human being. You have to help her’* (Participant, Kitongo Village). *‘They say: ´Push! Push! It is your problem if you kill your child, it is your problem! Just push!”* (Participant, Kitongo Village).

Although most women agreed that being slapped during labour is a serious violation of human rights and common form of humiliation, some women justified the behaviour of HCWs. HCWs were perceived as doing everything needed to ensure a good outcome, not complying with their directions could result in the death of the new-born for which the woman herself was then to blame.

*‘It is not humiliation because they are saving the life of the child and you are refusing. So, it is not humiliation. […] You are being told how to prepare for birth. During ANC visits you are being informed, you have to do this and this. And when you are giving birth, you are being instructed by the doctor, do this and this. […] But if you are being informed that much and yet you don’t follow instruction, then it is your own problem now’* (Participant, Matale Village).

## Being informed and listened to—Autonomy

Most women indicated how valuable it was to be descriptively informed by HCWs about what to expect in labour. This can also be interpreted as a matter of respecting rather than belittling a woman’s experience of pain:

*‘When you go there, you have to be told everything about how things are going. You have to be informed. So, that you can know what is going on after this hour… And if they see you are somehow afraid, they have to advise you in a good way to make sure that you relax because giving birth is not an easy thing’* (Participant, Mahaha).

Women also clearly indicated that when it comes to interventions that may be necessary to ensure a safe birth process, they need to be actively involved in decision-making.

*‘If you don’t have any health problems [if you are conscious], then it is no good not to be informed. I have to decide whether to go or not, because I have to sign’* (Participant, Matale Village).

Women appeared to rely heavily on HCWs advice with regards to decision-making and respect their advice. Women did however express they felt HCWs did not always provide an environment where they could express their questions or concerns. Some women explicitly made it clear that although they believed they were ‘heard’ by HCWs, this did not necessarily equate with being ‘listened to’.

*‘Yes they hear you. But, to be heard doesn’t mean that they are going to act [on what you have asked them]’* (Participant, Mahaha Village).  *‘Maybe you think that something is good for you and that it is your right to have it. Then you can tell but they might not receive it, they might not react on it. You can just say whatever you want but they don’t react, they don’t receive it, they don’t accept it’* (Participant, Mahaha Village).

The hierarchy differences between HCWs and women, based on educational level or class contributed to the women’s silence. Some women explained that even when they thought they were right and the HCW was wrong, they kept silent and respected the decision of the HCW.

*‘Most of the time, women don’t talk. They know they have their rights but most of them they don’t talk because they just respect the HCW, they know that it is her work. […] You cannot say anything because she is doing her work. She is in her own territory. You cannot do anything’* (Participant, Matale Village).

Some women explained that a woman is not heard because they don’t know their rights. Others stated that women are sometimes afraid for the responses of the HCWs, as this may result in being scolded. As a consequence they prefer to keep silent and felt devoid of an effective voice.

*‘It depends, if the health care provider comes and is just scolding you, you cannot say anything because you make it worse. But if she comes and advise you, do this and this, you can talk. Maybe then they can understand each other. But if someone comes with high voice and scolding you, you just keep quiet. You are just humiliated but you keep quiet’ (Participant, Matale Village).*

Another reason for silence was perceived lack of privacy. Women explained that they often received treatment when other HCWs or patients were in the same room. When women are then asked about their problems, they were hindered to speak freely as privacy and confidentiality were not guaranteed.

*‘If you have a health problem and you tell the HCW so that she can help you. But instead, she just talks to other people. […] So, later everyone knows that you have this disease because it is spread by the health worker’* (Participant, Mahaha Village).

## Being treated equally—Equality

The perception of equality was ‘to receive the same kind of services as anybody else’. However, it was easier for women to talk about situations in which this principle of equality was violated. Discrimination was related to personal characteristics, age and experience, coming from rural or urban areas, as well as cleanliness, being known, religious beliefs and money.

*‘For example, when you pray in the same church, you’ll know each other. You are going to receive very good services. […] You know sometimes, people they are afraid to ask which religion you practise. To find out, not by asking directly, he says: ‘Your patient needs prayer. So, we have to pray for her.’ Then they will find out. For example for Muslims, it is very bad nowadays’ (Participant, Mahaha Village).  ‘Cleanliness is important because it can reduce discrimination’* (Participant, Magu Town)*.*

In addition to long waiting times, women from rural areas reported that they were scolded. For example, one woman explained how these women are scolded for dressing their babies in old and unclean clothes.

*‘For example, they want to treat your baby only when she is wearing clean clothes. But here in the villages they don’t have that, they cannot afford that. They just take their child there. But when you go there, you just be scolded, scolded’* (Participant, Matale Village).

Women indicated that poverty could lead to abusive behaviour of HCWs due to the fact that not all people are able to purchase all needed supplies for birth at the health facilities. Women explained that during antenatal care HCW explain that due to lack of resources women are expected to bring their own supplies such as gloves, syringes, stitches and sometimes even kerosene. Women who are unable to bring it might be confronted with abusive behaviour of HCWs such as having to wait a long time or being scolded at.

*‘[For] the deliveries, they [women] were asked to pay money. If they don’t have they are told: ‘that husband of yours who has given you that pregnancy, he cannot give you money?’ (Participant Kitongo Village).*

## Feeling safe—Security

Most women indicated that they felt safe to give birth in a health facility. This feeling of being safe was often strongly associated with ‘the outcome of birth’.

*‘I was thankful because I was operated well and I was safe. So, if it would have been different, then I would say that it was not safe but for me it was safe and sound’* (Participant, Matale Village).

Women did however describe they experienced or heard about several unsafe situations which can occur during labour. These situations were related to labour complications, such as severe loss of blood, cord or placenta problems, convulsions or mal-presentation of the baby. All examples forming strong arguments for skilled attendance at birth which increases women’s experience of safety.

*‘Sometimes when a woman delivers, the baby comes out with cord around him. So, if she is alone and the baby is covered by that, the baby might die. But if someone is there to help, it would be good [and safe]’* (Participant, Matale Village).

Also examples were given of unsafe conditions in the facility related to lack of human resources or necessary supplies. The latter included examples of women being left alone to give birth, were refused services or where lack of available equipment resulted in unfavourable outcomes

*‘You can go there and be received but after that they don’t care. They are just walking around and you are in labour. Sometimes it leads you to deliver on your own. […] So there is no need even of going to the hospital. It is better you deliver at home because there is no help. You went there but you delivered alone’* (Participant, Mahaha Village).

Despite previous accounts of women valuing privacy, some women described how they preferred to give birth with multiple women in the same room if that would guarantee proximity of HCWs, which would increase chances of being helped on time.

### Does it matter?

Although human rights and its principles remained abstract terms, women were able to express the meaning of respect for and violation of these principles in the context of their experiences with institutionalized maternity care. Frequent accounts of violations of their rights lead to the question if this influenced their health seeking behaviour.

The majority of the participants favoured giving birth in a health facility because this would offer opportunities for assistance in case complications arise, which are often not available at home. Also, women appeared confident about HCWs’ knowledge and expertise in spite of their reported negative experiences concerning HCW behaviour. In the end, receiving *some* services appears better than no services at all.

*‘It’s not good to give birth at home; you have to go to the dispensary. You have to accept the services that you are going to receive. You cannot compare like home because you can have like problems, when giving birth. Like bleeding continuously. So it’s better you can get some help there’* (Participant, Mahaha Village).  ‘*All of my children I gave birth at the dispensary. I must go to the services, even though it is bad. When that day is bad, it is just bad luck***’** (Participant, Mahaha Village).

Although experiences of the interviewed women highlights they know how they prefer services to be, they explained some women might consider services, which violate some of the human rights principles, as ‘normal’ because of lack of knowledge.

*‘But there are some people; they take it like it is normal. Maybe they don’t know if this is their right and the others just get used to it, when I go there, I am not going to get the services’* (Participant, Mahaha Village).

One woman stated that if women know their rights they have more opportunity to assert demands for the provision of dignified services that she is entitled to.

‘*Currently, women are being humiliated and they don’t know where to complain and to find their rights. […] So, if you educate them and they are becoming knowledgeable about their rights, they will know where to go and claim their human rights‘* (Participant, Magu Town).

However, it remains a challenge, as women are afraid the situation may deteriorate when they show disagreement with HCW’s, as they fear repercussions. They appear to try to balance the way they are treated with the consequences of doing something about it and thus exercise patience.

*‘Ah no… You don’t accept to be treated like that, but you see as the days go by, maybe there is 1 day you are going to be treated in a good way. You just leave it to God. That is the circle of life. There are good things, there are bad things. You just be patient and see how it will go. You don’t like to be treated like that. But you have to be patient; you have to be patient’* (Participant, Magu Town).

## Discussion

This study explored the local, contextual meaning of human rights principles through accounts of women’s experiences and perceptions of maternity care. Women were able to convey how they thought they should be treated and how they should not be treated according to these principles when seeking maternal health services. Women explained that being treated with respect for dignity meant ‘being treated well’ regardless of any personal characteristics such as religion or economic status. Some women were fully aware that their rights were being violated.

Although most women might not know what to expect or what care should be provided during pregnancy and childbirth they are able to express if they are satisfied with the services or not and what this implies [6, 49]. Similar to previous studies in sub-Sahara Africa this research highlights women understand human rights ’to be equal’ and ‘to be received well’ by HCWs [6, 50]. In 2000 the UN committee on economic social and cultural rights adopted a general comment to the right to health to clarify the provision in article 12 of the ICESCR: ‘The right to health […] contains the following interrelated and essential elements [….] availability […] accessibility […] has four overlapping dimensions […] non discrimination […] physical accessibility […] affordability […] information accessibility […] acceptability […] quality [51]. For maternity care women in our study explained this to simply mean; to be greeted with a smile and calm words, assigned a bed if needed, being provided with basic medicines and supplies and having the ability to ask questions and be heard.

Women often talked about human rights principles in terms of violations caused by the attitudes of HCWs. This suggests that HCWs’ behaviour such as abusive language and practices is a major contributor to women’s perceptions of care, similar to previous findings [5, 6, 22, 49, 50, 52, 53]. Women described that although HCWs could sometimes show a disrespectful attitude this could also be the result of the patients own behaviour, and therefore should not be seen as intentionally humiliating. Additionally, disrespectful behaviour can be attributed to organizational issues in the health facility such as lack of staff, underpayment of staff, insufficient space and poor resource allocation [54]. The interpretation of slapping women during labour as a form of quality care has been supported in a previous study by Brown who states, “I do not mean to belittle patients’ complaints, nor to excuse malpractice and cruelty, but rather to underline convergences between caring relationships … that elucidate more complex understandings of what constitutes care in this setting [55]”. One understanding elucidated in that study was that for some HCWs to sympathise or pamper labouring women would result in them ‘relaxing’ and in doing so would hinder the progression of labour and endanger both mother and baby. Women are admonished if they make “too much noise” or if they don’t comply with health workers instructions when it is time to push. Firm actions become socially approved as failure to comply might result in death of the child [56]. Context is therefor important to understand how dignity encounters evolve and how they give meaning to individuals.

Acceptance of negative attitudes and practices of health professionals also reflect the institutional and hierarchical organisation of health facilities as well as the general social construction of gender and power imbalances [5, 57]. From a social norms perspective pregnant women from rural areas could act submissive or normalize how they are treated based on perceived expectations of the society or out of fear that failure to act according to these expectations will lead to social sanctions [58]. Having the ‘HCW role’ gives HCWs power over pregnant women with expectations of authority and knowledge, consequently women are heavily dependant on these HCWs and willingly trust and comply with their practices. Lacking reference as to what to expect during labour and being alone and dependent on the available HCWs, hardly facilitates opportunities for women to make an informed choice. HCWs that perform harmful behaviour, knowingly or unknowingly, provide women with the impression that they know what they are doing and that it is ‘correct behaviour in their given situation’ or there was nothing else they could have done.

As many women remain silent about their dissatisfaction with the sub-standard quality of care, or do not have a clear idea about what to expect, it is reasonable to assume they submit to the situation they are in. A number of studies have portrayed women as submissive in relation to the care they receive and ignorant of their rights [3, 5, 6, 52]. However, this research found that women do not easily submit themselves to sub-standard treatment. In particular when they are confronted with undignified and unequal treatment, they appear resilient, find ways to cope or adapt and are patient. Although they don’t demand better services they voice their disapproval of how services are currently being provided when asked directly, like in this study. However, it goes without saying, this is when looking *back* at childbirth experiences, and not *during* birth. Kabakian-Khasholian et al*.* [52] argued that women from rural areas are less demanding because of their low educational level and knowledge of other kinds of treatment. Kumbani et al*.* [52] stated that most women are unaware of the respectful care that they should receive because they do not know their rights and this in turn limits access to maternal health services. However, women have vast experience and expectations of social conduct, appropriate behaviour, politeness and cultural ways of dealing with others [6]. In this study it is possible women’s limited choice of alternatives for seeking maternal health services contributes to their silence. Women cope with the sub-standard treatment because of limited alternatives and the idea that *some* care is better than no care at all, rather than because of ‘*limited understanding’* [6]. They are however hindered in claiming their rights and lack agency in demanding better care.

Several groups and organizations are currently underway to develop and test interventions to reduce incidences of disrespect and abuse during childbirth [59]. This research can inform current efforts to promote respectful maternity care and illustrates how women’s views of health services can be instrumental in conceptualizing complex quality of care concepts. Interventions should be adapted to included contextual understanding of what constitutes both respectful and disrespectful care, according to women and health workers [60]. This study also shows, that while it appears easier to reflect on human rights principles in terms of violations, it is important that promotion of respectful care includes conceptualization and understanding of lived experiences of dignified and respectful care, beyond focus on harmful practices only. Besides experiences of violations, women presented examples of how health workers provided them with good and respectful services or showed appreciation of health workers’ technical skills to save their lives and that of their newborns. Health workers can sing a song to labouring women for comfort, while at the same time violating women’s privacy by leaving them unnecessarily uncovered. Dichotomous quality categories cannot capture the complexity of health worker behaviour and interaction with their patients [61]. Qualitative social studies therefor remain essential to contribute to the continuous efforts to measure quality of care in particular with regards to the interpersonal quality of care [14, 62]. Further research regarding health workers views and perceptions of how human rights principles can be incorporated in their daily work practices, within their complex environment is essential for further understanding of the occurrence of substandard care and to ensure interventions to promote respectful maternity care will have the desired effects.

### Study limitations

Limitations may have biased the eventual results of this study. First of all, this study is based on a small sample size of 17 women in a particular age group. Findings are therefore not necessarily representatives for younger pregnant women. Additionally, the topic’s sensitivity might have hindered women from expressing themselves freely. Women might not have told their most relevant experiences in order to protect themselves from pain or shame when memorizing the situation they were in. In order to build rapport with the participants, multiple interviews were conducted per woman during this study. Regardless of this, the language barrier remained a major challenge in upholding the quality of the data. During the interviews and FGD a translator was essential, also for interpretation of cultural and local habits. As the findings of this study are especially dependent on the exact words a woman used when expressing herself, it is likely that the use of a translator biased the eventual findings. Finally, as women were selected with help of the village executive officer, a selection bias might be present as a consequence of the hierarchical structures of the community, for example, the leader might have only selected women he knew or who were most vocal about their opinion of maternal health services.

## Conclusion

All people are entitled to a full set of human rights and its inherent principles, with dignity as the core principle. However, the substantive meanings of human rights principles (i.e. dignity, autonomy, safety, and equality) are often far from clear and are strongly interrelated. This research took a step toward understanding these principles by studying women’s experiences and perceptions, which resulted in numerous exemplified situations. Women experienced or witnessed a variety of situations in which human rights principles were either respected or violated. Women are able to assess the provided maternal health services and appear to be well aware how they should and should not be treated according to their own understanding of human rights. Although women strongly condemn abusive behaviour of health workers, they appear to understand this to be in their best interest or endure it in order to adhere to social expectations or out of lack of alternatives.

## Abbreviations

HCWs, heath care workers; ICESCR, The International Covenant on Economic, Social and Cultural Rights; UDHR, United Declaration of Human Rights

## Additional file

Additional file 1: Interview Guide. The interview guide includes examples of questions asked to participants. (DOCX 17 kb)
