# Supplementary material for: Maternity care and Human Rights: what do women think?
Source: BMC Int Health Hum Rights. 2016 Jul 2;16:17. doi: 10.1186/s12914-016-0091-1 (PMC4930607; doi:10.1186/s12914-016-0091-1)
Supplement: Additional file 1: — Interview Guide. The interview guide includes examples of questions asked to participants. (DOCX 17 kb) [file 12914_2016_91_MOESM1_ESM.docx]

**(Semi Structured) In-depth Interview Guideline**

**Introduction**

Thank you for taking your time to meet me today. My name is Fleur (Flora) and I am a student from the Netherlands. For my master study I conduct research on maternal health in Magu District in collaboration with CROMABU, a crops marketing bureau in Magu Town. For this research I am interested in your experiences and perceptions of human rights principles in terms of (received) maternal health services. I would like to discuss this with you as you probably posses life experience and specific knowledge that I as a researcher do not have. This interview consists of two parts and each will take about one hour. With your permission I would like to audio-recorder the interview because I do not want to miss any of your comments. [Turn audio recording device on] All responses will be kept confidential. This means that your interview responses will only be shared with research team members. Any personal information in the final report will not identify you as the respondent. Furthermore, the recording will also be deleted when reports have been written. We hope that this research with your help will benefit the women in the community. However, for yourself personally there will be no benefits but also no risk of taking part in the research. I emphasise that you do not have to talk about anything you do not want to. You can stop participation any time and without any explanation. Do you have any questions about this explanation? Are you willing to participate? If you have any questions during our conversations, please do not hesitate to ask.

**Start Interview**

***Human Rights Principles***

- Kindly please, can you explain what human rights are to you?

*Probing Questions*

- - What exactly do mean with that?
- What does that [*answer given*] mean to you?
  - What does dignity mean to you?
  - What does autonomy mean to you?
  - What does security mean to you?
  - What does equality mean to you?
- Kindly please, how did you experience this [*answer given*] in your personal life? Can you give an example/experience of your personal life?
- Can you explain what exactly made you feel like that [*answer given*]?

*Probing Questions*

- - Why did you feel like that?
  - Can you describe the situation?
  - Can you describe the setting?
  - Can you describe the interaction with other persons?
  - What of this made you feel like that [*answer given*]?

***Link to Maternal Health***

Like I explained before, I am interested in the meaning of these principles in the maternal health setting.

- Kindly please, can you describe your pregnancy and/or labour?
- Kindly please, how did you experience this [*answer given*] during pregnancy and/or labour? Can you give an example/experience?
- Can you explain what exactly made you feel like that [*answer given*]?

*Probing Questions:*

- - Why did you feel like that?
  - Who accompanied you during your visit to the health clinic?
  - Can you describe the surrounding of the health facility?
  - How many people were at the health facility?
  - How was your interaction with the health professionals?
  - Can you describe the attitudes and practices of health professionals?
  - What of this made you feel like that [*answer given*]?
  - Best/Worst experience?

***Women’s Recommendations***

- According to your opinion, what should be changed in health facilities in order to ensure you are treated with [*answer given*], dignity, autonomy, security, and equality?

**End interview**

Do you have any additional thoughts you would like to share? Or do you have any comments or questions concerning the interview?

Thank you for your time.
